# Maternal High Fat Diet Programs Male Mice Offspring Hyperphagia and Obesity: Mechanism of Increased Appetite Neurons via Altered Neurogenic Factors and Nutrient Sensor AMPK

**DOI:** 10.3390/nu12113326

**Published:** 2020-10-29

**Authors:** Mina Desai, Monica G. Ferrini, Guang Han, Kavita Narwani, Michael G. Ross

**Affiliations:** 1 Department of Obstetrics and Gynecology, Perinatal Research Laboratory, The Lundquist Institute at Harbor-UCLA Medical Center, Torrance, CA 90502, USA; ghan@lundquist.org (G.H.); knarwani@lundquist.org (K.N.); mikeross@ucla.edu (M.G.R.); 2 Department of Obstetrics and Gynecology, David Geffen School of Medicine, University of California, Los Angeles, CA 90024, USA; 3 Department of Health and Life Sciences, Charles R. Drew University, Los Angeles, CA 90059, USA; monicaferrini@cdrewu.edu; 4 Department of Internal Medicine, Charles R. Drew University, Los Angeles, CA 90059, USA; 5 Department of Obstetrics and Gynecology, Charles R. Drew University, Los Angeles, CA 90059, USA

**Keywords:** developmental programming, neuropeptides, hypothalamic arcuate nucleus, neuroprogenitor cells, AMPK activator

## Abstract

Maternal high-fat (HF) is associated with offspring hyperphagia and obesity. We hypothesized that maternal HF alters fetal neuroprogenitor cell (NPC) and hypothalamic arcuate nucleus (ARC) development with preferential differentiation of neurons towards orexigenic (NPY/AgRP) versus anorexigenic (POMC) neurons, leading to offspring hyperphagia and obesity. Furthermore, these changes may involve hypothalamic bHLH neuroregulatory factors (Hes1, Mash1, Ngn3) and energy sensor AMPK. Female mice were fed either a control or a high fat (HF) diet prior to mating, and during pregnancy and lactation. HF male newborns were heavier at birth and exhibited decreased protein expression of hypothalamic bHLH factors, pAMPK/AMPK and POMC with increased AgRP. As adults, these changes persisted though with increased ARC pAMPK/AMPK. Importantly, the total NPY neurons were increased, which was consistent with the increased food intake and adult fat mass. Further, NPCs from HF newborn hypothalamic tissue showed similar changes with preferential NPC neuronal differentiation towards NPY. Lastly, the role of AMPK was further confirmed with in vitro treatment of Control NPCs with pharmacologic AMPK modulators. Thus, the altered ARC development of HF offspring results in excess appetite and reduced satiety leading to obesity. The underlying mechanism may involve AMPK/bHLH pathways.

## 1. Introduction

Obesity and its related diseases are the leading cause of death in Western society. Currently, more than two-thirds of adults in the United States are overweight and over one-third are obese [1]. Increased energy intake, not reduced energy expenditure, likely explains population weight gain in US children and adults [2,3]. Despite this understanding, surprisingly little is known as to why the energy intake is increased in select individuals.

The intrauterine environment contributes significantly to the propensity for offspring obesity. There is a marked risk for programmed adult metabolic syndrome in relation to birthweight [4,5], with both small- and large-for-gestational-age-infants at risk of programmed obesity [6]. In women, maternal obesity is associated with macrosomia [7], childhood obesity and offspring metabolic syndrome [8]. Animal studies have replicated human evidence of programmed obesity. In mice, naturally occurring neonatal macrosomia is a risk factor of adult metabolic syndrome [9], while in rats, offspring of obese and high-fat diet-fed dams are predisposed to adult obesity when fed a postnatal high fat (HF) diet [10].

Appetite is primarily controlled by a circuit of hypothalamic nuclei. The predominant appetite regulatory site, the arcuate nucleus (ARC), contains two populations of neurons with opposing actions on food intake: primarily medial ARC appetite/orexigenic (Neuropeptide Y (NPY) and agouti-related protein (AgRP)) and primarily lateral ARC satiety/anorexigenic (proopiomelanocortin (POMC) and cocaine and amphetamine-regulated transcript (CART)) neurons. Even moderate overexpression of ARC NPY, and likely underexpression of POMC, is sufficient to induce overfeeding and obesity [11].

Key hypothalamic nuclei begin to be populated during fetal life, with continued neural development and remodeling during the neonatal period [12]. In rodents, nonhuman primates and humans, neurogenesis primarily occurs during early to mid-gestation. The postnatal hypothalamic axon growth in rodents, however, differs from that in nonhuman primates and humans, where hypothalamic neural projections develop almost entirely during fetal life [13]. During development, hypothalamic neuroprogenitor cells (NPCs) in the peri-third ventricular zone undergo extensive proliferation, self-renewal, and ultimate terminal division into cells destined for neuronal or glial fate [14]. Following terminal division, these neurons migrate to ultimate nuclei sites, differentiate to specific neuronal phenotypes, and form functional circuits. NPCs differentiated to neurons destined for the ARC further differentiate to orexigenic (NPY) or anorexigenic (POMC) phenotypes [15,16]. NPC proliferation and differentiation processes are regulated by a series of neuroregulatory basic helix-loop-helix (bHLH) transcription factors, including Hes1, Neurogenin (Ngn3), and Mash1 [17]. Hes1 is a transcriptional repressor of genes and acts to maintain proliferating NPCs. In mice with brain specific Hes1-deficiency, NPCs prematurely differentiate [18], reducing the NPC pool. Both Ngn3 and Mash1 are required for normal development of POMC neurons [19,20], while Ngn3 also inhibits NPY expression [21]. Thus, Ngn3(-/-) mice express markedly reduced POMC [21] and an increased number of NPY cells [22]. These bHLH factors exhibit an established spatial/temporal pattern, which controls NPC differentiation, but may be altered in response to energy/nutrient factors, including 5′ AMP-activated protein kinase (AMPK), a heterotrimer complex that is activated (i.e., phosphorylated) in response to reduced energy levels.

We hypothesized that offspring of obese, high-fat-diet-fed dams exhibit obesity secondary to programmed hyperphagia, and that this results from enhanced development of appetite and impaired development of satiety neurons. We studied the changes both in vivo (hypothalamic tissue) and in vitro (NPCs) of bHLH factors, neuropeptide and AMPK expression, including neuronal sub-type in offspring exposed to maternal obesity and HF diet.

## 2. Materials and Methods

### 2.1. Ethical Approval

The study was approved by the Institutional Animal Care and Use Committee at The Lundquist Institute at Harbor UCLA (formerly known as the Los Angles Biomedical Research Institute; Project No. 12503) and was conducted in strict accordance with guidelines provided by the American Accreditation Association of Laboratory Care and the Public Health Service Policy on Humane Care and Use of Laboratory Animals, and conforms to the principles and regulations as described in the Editorial by Grundy [23].

### 2.2. Maternal Diet

Weanling female C57BL/6 mice purchased from Jackson Laboratory (Bar Harbor, ME, USA) were housed at constant temperature and humidity, on a controlled 12 h light/dark cycle with free access to food and drinking water. Mice were randomly assigned to a high fat diet (*N* = 12, 45% Kcal; Research Diet D12451, New Brunswick, NJ, USA) to create obesity prior to mating, or a control diet (*N* = 12, 10% Kcal; Research Diet D12450B, New Brunswick, NJ, USA). At 11 weeks of age, females from both groups were mated and maintained on the same diet during pregnancy and lactation. Animals gave birth spontaneously.

### 2.3. Offspring Studies

Two groups of offspring were studied, HF offspring (born to dams fed a HF diet during pregnancy and lactation and thereafter provided a normal fat diet) and control offspring (born to Control dams fed a normal fat diet throughout the study period). It should be noted that the studies were undertaken only in males as estrogen is known to influence food intake [24,25], which would entail proestrus confirmation in females prior to tissue retrieval.

For studies of newborns at postnatal one day of age (p1), *N* = 6 litters per group were used. From each litter, four male pups were euthanized by rapid decapitation, brain removed and hypothalami dissected and used for analysis (due to difficulty in accurately dissecting the ARC). In each case, hypothalami from one male pup were snap frozen and stored at −80 ℃ for protein expression analysis. Hypothalami from the remaining three male pups were pooled for NPC cultures.

For long-term studies till 12 months of age (to evaluate persistence of changes), separate litters of *N* = 6 per group were used. Following birth, litter size at postnatal age p1 was standardized to three males and three females (to normalize nursing), and all offspring were weaned to control 10% Kcal diet at p21 till 12 months of age. At 3 weeks of age, pups were housed separately and weekly body weights were recorded. Food intake was measured daily by providing a weighed amount every morning and after 24 h, weighing the amount of food remaining, including any on the bottom of the cages. Intake was calculated as the weight (in grams) of food provided less that recovered and average obtained for the week.

At 12 months of age, six males (one per litter per group) underwent non-invasive DEXA scan. Mice were anesthetized (ketamine 90 mg/kg body weight, i.p and xylazine 10 mg/kg body weight, i.p) and placed in a micro-isolator cage with warm water bottles to avoid hypothermia. After 48 h recovery, males were euthanized by inhalation of isoflurane (5% in chamber and 2% via mask) followed by exsanguination via cardiac puncture and decapitation. Blood was collected in heparinized tubes for plasma analysis of leptin levels (mice-specific ELISA assay kit #AB0334, Sigma, St. Louis, MO, USA), and brain removed, ARC dissected, snap frozen, and stored at −80 ℃ for protein expression analysis. At 12 months of age, an additional six males (one per litter per group) were anesthetized (ketamine 90 mg/kg body weight, i.p.; xylazine 10 mg/kg body weight, i.p.) and intracardially perfused with saline followed by 4% paraformaldehyde [26] brains collected and processed for neuronal count.

### 2.4. In Vitro Treatment with AMPK Modulators

To further explore AMPK-mediated effects, NPCs from postnatal 1-day old (p1) Control newborn males were cultured in differentiating medium and treated in vitro with AMPK activator (AICAR; 0.25, 1, 2 mM; #A9978, Sigma-Aldrich, St. Louis, MO, USA), AMPK inhibitor (1, 5, 25 µM Dorsomorphin dihydrochloride, Compound C BML-275,#21207, Cayman Chemicals, Ann Arbor, MI, USA) or vehicle. Compound C (BML-275) is a potent, reversible, selective AMPK inhibitor with Ki of 109 nM in cell-free assays, exhibiting no significant inhibition of several structurally-related kinases including ZAPK, SYK, PKCθ, PKA, and JAK3 [27].

### 2.5. Hypothalamus/ARC Dissection

Regions on the two sides of hypothalamus were cut, the dorsal part removed, and the ventral part was retrieved for study. For ARC microdissection, the area adjacent to the bottom of the third ventricle was dissected parallel to the border of the ventricle using the fornix and third ventricle as landmarks [21,28].

### 2.6. Tissue Neuronal and NPY Counts

Fixed brains from 12-month-old males were sectioned (30µm) between Bregma 1.67–1.79 mm. Every third section was immunostained for NPY (1:500, Abcam, Cambridge, MA, USA) and Tuj1 (1:500, Sigma-Aldrich, St. Louis, MO, USA) and visualized with donkey anti-goat-IgG-Alexa 568 and donkey anti-rabbit-IgG-Alexa 488. Photomicrographs were captured at 20× magnification using computer-assisted florescence microscopy (Zeiss, Axioskop 40, San Diego, CA, USA) for unbiased quantification of positive labeled cells. Image J software (https://imagej.nih.gov/ij/index.html, NIH, Washington DC, USA) was used to determine total neuronal and NPY counts [28,29,30]. Briefly, the following settings were used for NPY counts: Adjustments-image ➔ split channels ➔ (8 bit) ➔ Adjust ➔ Threshold (dark background). Size (pixel^2^): 20× (50-Infinity) and circularity (0.2–1.00) were used for subsequent measurements and analysis. In each case, six sections were counted per animal.

### 2.7. Neurosphere Cultures

NPCs cultures were prepared from hypothalamic periventricular sections as previously reported [31,32]. Briefly, hypothalamus was dissected in DMEM/F12 medium, cells disassociated by trypsin, centrifuged and cells seeded at 5 × 10^4^ cell/mL in complete medium [NeurobasalTM Medium containing 1% anti-anti (Invitrogen, Waltham, MA, USA), 2% B27 (Cat#17504-044, GIBCO, Waltham, MA, USA), 20 ng/mL FGF2 (Sigma-Aldrich), 20 ng/mL EGF (Sigma-Aldrich), 1 μg/mL heparin (Eli Lilly, Indianapolis, IN, USA), and 2.5 μg/mL L-glutamine (Invitrogen)]. After 8 days in culture (passage 0), centrifuged neurospheres were dissociated into single-cell by trypsinization and reseeded at same cell density (passage 1) in complete medium. For induction of differentiation, dissociated cells were re-suspended in differentiating medium (in absence of FGF2, EGF and heparin) and seeded in culture dishes pre-coated with 0.01% poly-L-lysine (Sigma-Aldrich). 

For protein expression analysis, the disassociated neurosphere cells in complete or differentiating medium were seeded in 6-well plates. At day 8 of culture, the cells were harvested and dissolved in RIPA solution (Cell Signaling, Beverly, MA, USA) with protease inhibitor cocktail (Thermo Fisher Scientific, Irwindale, CA, USA), briefly sonicated and the cell lysates processed for determination of protein content of by BAC™ Protein Assay Kit (Thermo Fisher Scientific).

For NPY neuronal count, cells were cultured in differentiating medium for 8 days and subsequently fixed in 4% paraformaldehyde in PBS and immunostained with rabbit anti-NPY (1:500, Abcam,). Goat anti-rabbit IgG-Alexa 488 was used to visualize positive cells. All cells were stained by DAPI. In each case, 1000 DAPI cells were counted and positive labeled cells were counted using Image J program.

### 2.8. Western Blot

Westerns were performed and normalized against GAPDH (Glyceraldehyde 3-phosphate dehydrogenase, 37 kd, 1:10,000, MAB374, Millipore, Billerica, MA, USA) as previously reported by our group [32]. For detection of pAMPK, NaF (50 mM) was added to all buffers, which had been demonstrated to be an effective phosphoseryl and phosphothreonyl protein phosphatase inhibitor [33]. Antibodies were obtained from Santa Cruz Biotechnology, Inc (Santa Cruz, CA, USA) unless otherwise specified: AgRP (1:500, 14 kd, sc-50299); POMC (1:500, 30 kd, sc-20148); Hes1 (1:500, 35 kd, sc-25392); Mash1 (1:500, 30 kd, sc-13222); Ngn3 (1:1000, 23 kd, ab38548, Abcam, Cambridge, MA); and AMPK (1:1000, 63 kd, sc-19128). All values were normalized to GAPDH and presented as fold change.

### 2.9. Statistics

Sample size estimates were based on a power of 80% to detect 30% changes between groups (assuming an expected SD of 20% of mean values). This analysis results in six animals in each group. In all cases, the data were tested for normal distribution and all samples collected were coded to avoid any bias in analysis. NCSS statistical software (NCSS, Kaysville, UT, USA) was used for data analysis. Differences between HF and the Controls were compared using unpaired *t*-test (DEXA scan, protein expression, counts, and leptin levels) and repeated ANOVA (body weights, food intake).

## 3. Results

### 3.1. Maternal Body Weight

As a result of the 45% fat diet, HF dams were significantly heavier than controls at mating, term gestation and end of lactation (Table 1). However, the litter sizes of HF and control groups were similar, averaging approximately seven pups.

### 3.2. Offspring Phenotype

**Figure 1:**
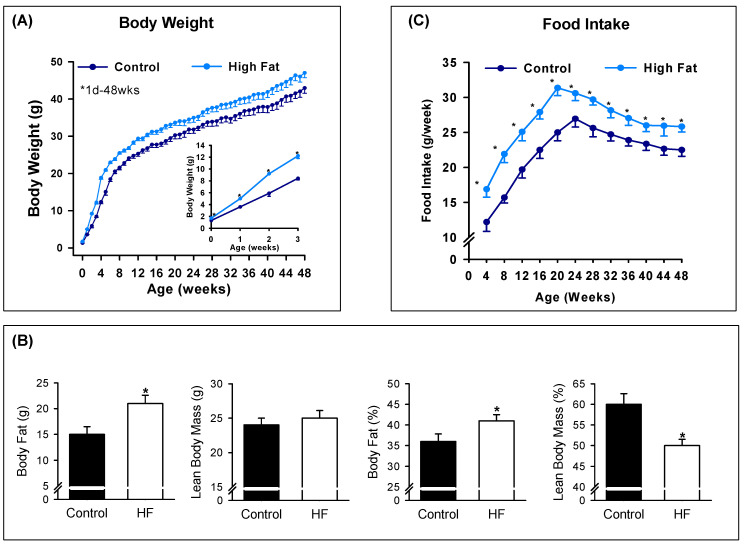
Maternal HF (high fat) increases male offspring body weight, adiposity and food intake. (**A**) Male offspring body weights from birth to 48 weeks (wks) of age of Controls (●) and HF (●); inset of body weights from 1 day to 3 weeks of age. (**B**) Body fat and lean body mass and percentage of body weight of 12-month old male offspring. (**C**) Food intake from 4 to 48 weeks of age of male offspring from control (●) and HF (●) groups. Values are mean ± SE of *n* = 6 males from six litters. * *p* < 0.01 vs control.

Maternal HF diet resulted in increased birth weight among male (1.60 ± 0.01 vs 1.31 ± 0.01 g; *p* < 0.05) newborns. HF offspring demonstrated further increase in body weight through the first 3 weeks of life with a continued acceleration of body weight through approximately 12 weeks of age. Thereafter, male HF offspring remained consistently heavier than controls with significantly increased percent and absolute body fat (g) at 12 months of age. Accordingly, HF male offspring demonstrated reduced percent lean body mass at 12 months of age, though with no change in absolute lean body mass (Figure 1A,B). Food intake of male offspring (beginning at weaning) was significantly greater than Controls throughout the first year of life (Figure 1C). Plasma leptin levels were significantly higher in 12-month-old HF males as compared to Controls (8.1 ± 1.2 vs 4.0 ± 0.8, *p* < 0.01).

### 3.3. Hypothalamic/ARC Protein Expression and Neuronal Counts

**Figure 2:**
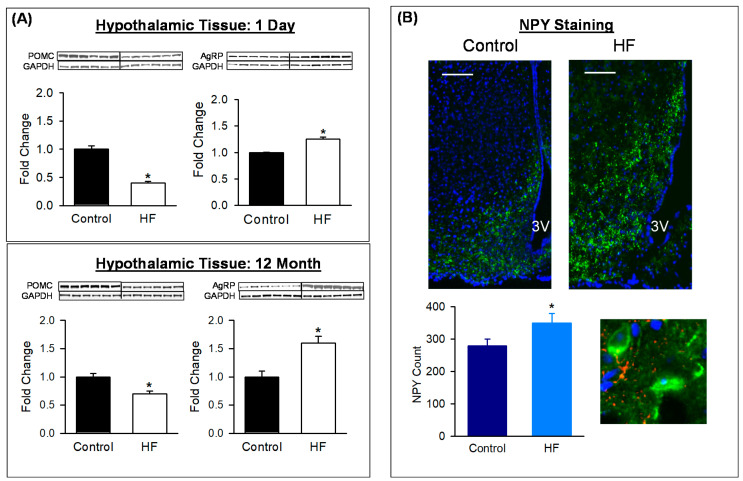
Maternal HF diet alters offspring hypothalamic tissue expression of neuropeptides and neuronal phenotype. (**A**) POMC and AgRP protein expression in hypothalamus of 1-day-old males, and ARC of 12-month-old males from Control (■) and HF (□) groups. Values are fold change (mean ± SE) of *n* = 6 males from six litters per group (1 male/litter). * *p* < 0.001 vs Control. (**B**) ARC sections from representative control (■) and HF (■) 12-month-old males, immunostained for NPY (green) at × 20 and × 100; scale bar = 50 µm. Total NPY (mean ± SE) of *n* = 6 males from six litters per group (1 male/litter). * *p* < 0.01 vs control. Abbreviations: HF (high fat); POMC (proopiomelanocortin); AgRP (agouti-related protein); NPY (neuropeptide Y); ARC (arcuate nucleus); 3V (third ventricle).

To investigate the underlying cause for hyperphagia, protein expression of hypothalamic/ARC neuropeptides were analyzed. In HF newborns at postnatal age p1, hypothalamic POMC expression was reduced while that of AgRP was increased. Similar to p1, at 12 months of age, ARC expression of POMC protein was reduced and AgRP increased (Figure 2A). When specific adult male neuronal counts were performed, HF adults demonstrated an increase in appetite (NPY) and neurons in the HF adults (Figure 2B).

**Figure 3:**
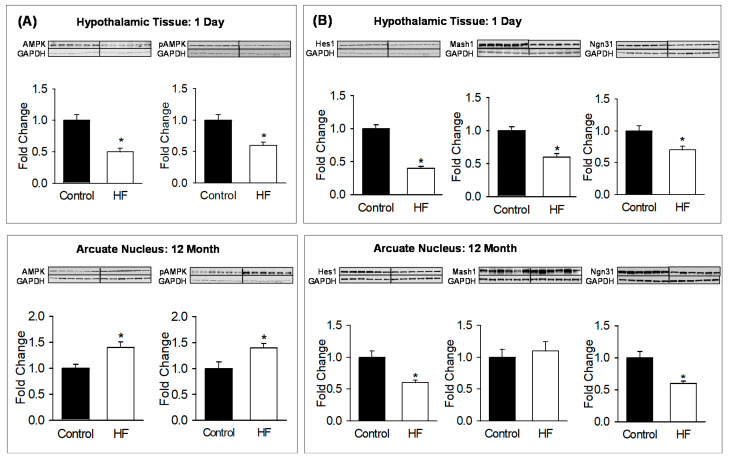
Maternal HF diet alters offspring hypothalamic tissue expression of AMPK, pAMPK and bHLH factors. (**A**) Hes1, Mash1, Ngn3, and (**B**) AMPK and pAMPK protein expression in hypothalami of 1-day-old males, and ARC of 12-month-old males from control (■) and HF (□) groups. Fold change (mean ± SE) of *n* = 6 males from six litters per group (1 male/litter). * *p* < 0.01 vs control. Abbreviations: HF (high fat); AMPK (5’ AMP-activated protein kinase); pAMPK (phosphorylated 5’ AMP-activated protein kinase); Hes1 (Hairy and Enhancer of Split 1); Mash1 (Achaete-scute complex homolog-1); Ngn3 (neurogenin 3); GAPDH (Glyceraldehyde 3-phosphate dehydrogenase).

To further analyze the underlying mechanism, hypothalamic protein expression of bHLH factors and metabolic sensor were measured. At postnatal age p1, HF newborns demonstrated a decrease in the expression of Hes1, Ngn3 and Mash1 (Figure 3A) with a reduction in hypothalamic tissue AMPK and pAMPK levels (Figure 3B). At 12 months of age, ARC protein expression showed persistent decrease in Hes1 and Ngn3 (Figure 3A) with increased AMPK and pAMPK levels (Figure 3B).

### 3.4. Hypothalamic Neuroprogenitor Cells

**Figure 4:**
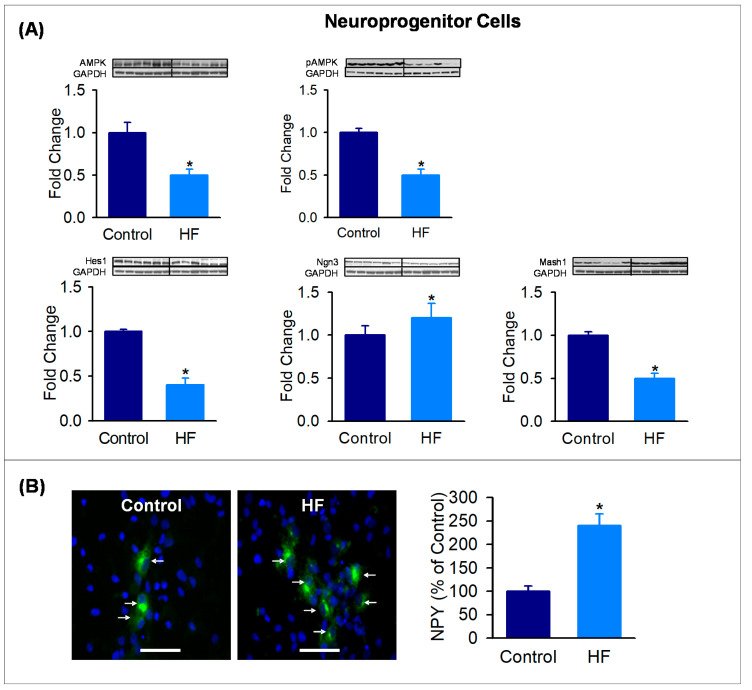
Maternal HF diet alters expression of AMPK, pAMPK and bHLH factors and promotes differentiation to NPY neurons in in vitro NPC culture. (**A**) NPC protein expression of AMPK, pAMPK and bHLH factors (Hes1, Mash1, Ngn3) in control (■) and HF (■) newborn males at 1 day of age. Fold change (mean ± SE) of *n* = 6 from six litters per group (1 male/litter). * *p* < 0.01 vs control. (**B**) NPCs from 1-day-old control (■) and HF (■) males were cultured in differentiating medium for 7 days, fixed and immunostained for NPY (green) and DAPI (blue). Scale bar = 50 µm values are percent of controls (mean ± SE). * *p* < 0.01 vs control. Abbreviations: HF (high fat); AMPK (5’ AMP-activated protein kinase); pAMPK (phosphorylated 5’ AMP-activated protein kinase); Hes1 (Hairy and Enhancer of Split 1); Mash1 (Achaete-scute complex homolog-1); Ngn3 (neurogenin 3); GAPDH (Glyceraldehyde 3-phosphate dehydrogenase); NPY (neuropeptide Y).

To explore the programming potential of neuroprogenitor cells, hypothalamic NPC cells were grown in culture from postnatal age p1 HF and Control offspring. In complete medium, NPCs cultured from HF newborns demonstrated significantly decreased Hes1 with reduced AMPK and pAMPK levels. In differentiating medium, NPCs cultured from HF newborns exhibited reduced Ngn3 and Mash1 (Figure 4A), consistent with hypothalamic tissue protein expression. When NPC were differentiated, HF NPCs demonstrated a marked increase in the differentiation to NPY neurons (Figure 4B).

### 3.5. In Vitro Treatment with AMPK Modulators

**Figure 5:**
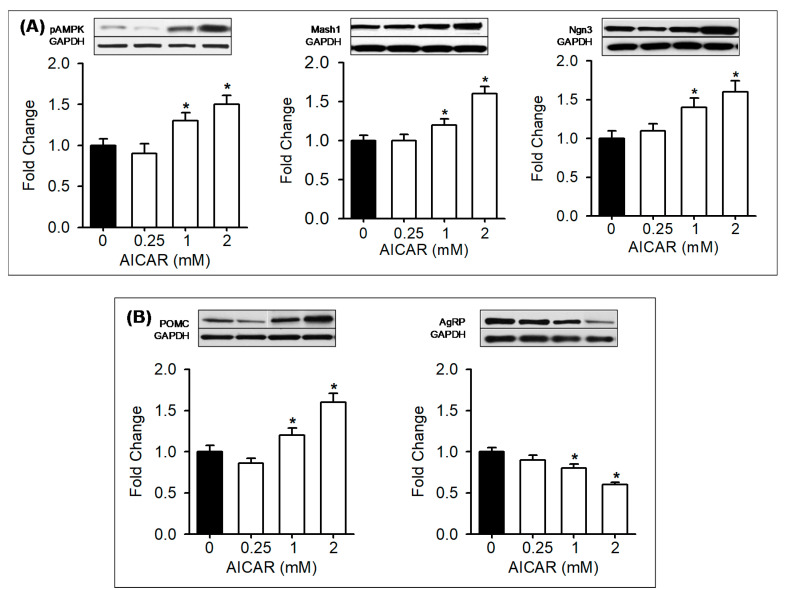
Activation of AMPK in vitro upregulates bHLH factors, suppresses appetite and increases POMC neuropeptide expression. (**A**) Control NPCs were treated in vitro with AMPK activator (0.5, 1, 2 mM; □) or vehicle (■). NPC protein expression with representative immunoblots of pAMPK, bHLH factors (Mash1, Ngn3) and (**B**) neuropeptides (POMC, AgRP). Values are fold change of vehicle treated (*N* = 5; mean ± SE). * *p* < 0.01 vs control. Abbreviations: AICAR (5-aminoimidazole-4-carboxamide-1-β-D-ribofuranoside); pAMPK (phosphorylated 5’ AMP-activated protein kinase); Mash1 (Achaete-scute complex homolog-1); Ngn3 (neurogenin 3); POMC (proopiomelanocortin); AgRP (agouti-related protein); GAPDH (Glyceraldehyde 3-phosphate dehydrogenase).

**Figure 6:**
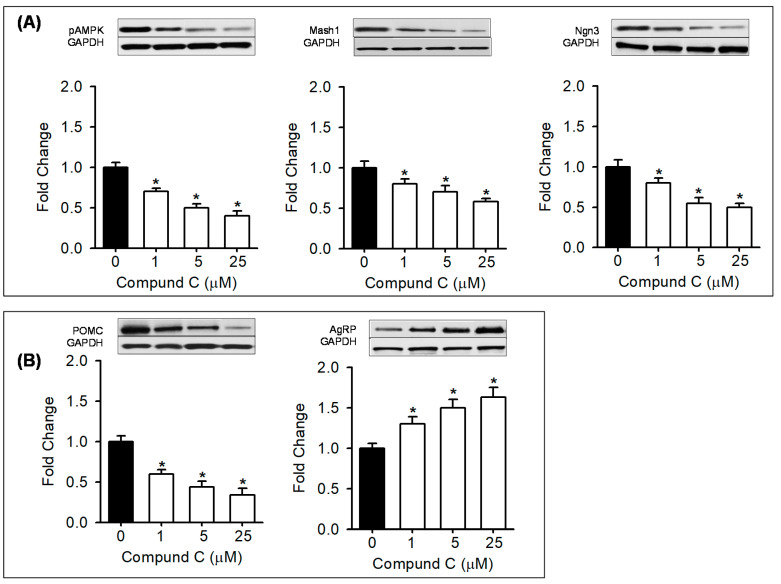
Suppression of AMPK in vitro downregulates bHLH factors and increases appetite and reduces satiety neuropeptide expression. (**A**) Control NPCs were treated in vitro with AMPK inhibitor (Compound C 1, 5, 25 µm; □) or vehicle (■). NPC protein expression with representative immunoblots of pAMPK, bHLH factors (Mash1, Ngn3) and (**B**) neuropeptides (POMC, AgRP). Values are fold change of vehicle treated (*N* = 5; mean ± SE). * *p* < 0.001 vs control. Abbreviations: Compound C (Dorsomorphin dihydrochloride); pAMPK (phosphorylated 5’ AMP-activated protein kinase); Mash1 (Achaete-scute complex homolog-1); Ngn3 (neurogenin 3); POMC (proopiomelanocortin); AgRP (agouti-related protein); GAPDH (Glyceraldehyde 3-phosphate dehydrogenase).

To further explore AMPK-mediated effects, hypothalamic NPCs from Control newborn males were treated in vitro with AMPK modulators. Control NPCs treated with AICAR at 1 and 2 mM doses showed increased pAMPK, Mash1, d Ngn3 and POMC with reduced AgRP (Figure 5A,B). Conversely, Control NPCs treated with Compound C showed a dose-dependent decrease in pAMPK, Mash1, and Ngn3 with decreased POMC and increased AgRP expression (Figure 6A,B).

## 4. Discussion

Consistent with prior reports [9,10,34,35], the present study demonstrates that maternal obesity and high-fat diet programs offspring hyperphagia and obesity. However, the current study is the first to demonstrate that the hyperphagia may be a result of NPCs preferentially differentiating to NPY neurons during development and ultimately leading to increased appetite (NPY) neurons as evident in the adult offspring in vivo. Further, these effects may be mediated by a pathway involving fetal hypothalamic nutrient sensor (AMPK) and neuroregulatory bHLH transcription factors. 

In the present study, offspring of HF dams demonstrated increased birth weight, similar to the increased prevalence of macrosomia seen among newborns of obese women [36,37] and animal model of dietary-induced obesity [10,34,35]. During the nursing period from birth to 21 days, the HF pups continued their trajectory of increased body weight gain. This may be in part due to increased milk intake, higher milk total caloric content of HF dams [38,39,40,41,42] and/or the direct consumption of HF diet prior to weaning. All these are likely to have a long-term impact on offspring energy metabolism. The HF offspring continued to be heavier with a significant increase in percent body fat, decrease in percent lean body mass and higher leptin levels than the controls. The accelerated body weight gain exhibited by the HF offspring was in parallel with their significantly increased food intake. The balance of appetite and satiety represents a critical regulator of food intake and obesity and individuals susceptible to obesity demonstrate a weak satiety response following meals [43]. In humans, infants born to obese mothers demonstrate increased weight gain [44,45] and more calorie consumption than those born to normal weight mothers [46,47]. Together, these findings are consistent with maternally-induced offspring obesity, mediated at least in part via programming of neuroprogenitor cells, which control appetite and food intake.

At 1 day postnatal and 12 months of age, HF male offspring demonstrated increased AgRP expression and reduced POMC expression in hypothalamic tissue and ARC. These findings suggest an imbalance in the appetite/satiety neuropeptides, which would promote hyperphagia. For example, newborn offspring of obese dams exhibit reduced hypothalamic POMC mRNA [48,49] and increased NPY signaling (PVN NPY1R) [50], and offspring of high-carbohydrate-fed dams demonstrate increased NPY release in the PVN [51].

However, as protein expression may not correlate with neuronal numbers, due to over- or under-expression of neuropeptides within individual neurons, we specifically examined the NPY neuronal counts. HF offspring demonstrated an increase in NPY neuronal count. Nonetheless, this finding needs to be confirmed by detailed robust analysis that includes measurements of cell size and volume.

To explore the mechanism for the altered ARC anatomy and neuropeptide expression, we examined the expression of key bHLH neurogenic factors at both ages. HF newborns demonstrated a reduction in hypothalamic tissue Hes1, which acts to maintain proliferating NPCs and suppress proneurogenic genes at both transcriptional and posttranscriptional levels [17,52], including protein stability [53]. In mice with brain-specific Hes1-deficiency, NPCs prematurely differentiate [18], reducing the NPC pool. The reduced Mash1 and Ngn3 at both 1 day postnatal and 12 months of age are consistent with the increased NPY neurons [18]. Together these findings suggest that a programmed alteration in neurogenic bHLH factors is likely responsible in part for the altered development of the ARC nucleus in HF offspring.

We further explored the role of the energy sensor AMPK. A high-fat diet decreases AMPK in systemic tissues [54], consistent with its role as a regulator of whole body energy balance [55]. AMPK monitors cellular energy status, becoming phosphorylated (pAMPK) in response to metabolic stresses that decrease ATP. Although an array of extracellular factors (e.g., leptin, insulin, IGF1) modulate NPC function, emerging evidence indicates that energy metabolism is a critical regulator of NPC proliferation/differentiation. During fetal development, induction of pAMPK potentiates NPC proliferation as well as neurogenesis [56]. Accordingly, AMPK β-subunit KO (AMPK β-/-) in mice results in reduced NPC proliferation [57] and in Drosophila causes extensive neurodegeneration [58]. The reduced level of hypothalamic AMPK and pAMPK in HF offspring at 1 day of age postnatal suggests relatively elevated energy levels in fetal brain resulting from maternal obesity and high fat diet. Although the pathway by which AMPK may regulate bHLH factors and/or neurodifferentiation is unclear, the present results suggest that during early life, energy sensing in the hypothalamus via AMPK may alter the NPY/POMC neuronal ratio.

We further examined the programmed potential of NPC cells removed from the HF newborn environment and grown in cell culture. Consistent with newborn hypothalamic tissue expression, NPC cells demonstrated significantly reduced AMPK and pAMPK expression. As the neuronal culture medium was identical in both HF and control groups, these findings would suggest a programmed down regulation of AMPK and pAMPK expression, independent of the energy composition of the culture media. Similarly, NPCs in culture demonstrated reduced Hes1, Mash1and Ngn3, simulating the changes seen in hypothalamic tissue of HF newborns. Importantly, when cultured in differentiating media, NPCs from HF newborns demonstrated nearly 2.5-fold increase in NPY neurons as compared to controls. These findings indicate that the complex pathway of neuroproliferation and NPC differentiation to neurons and ultimately neuronal expression of specific neuropeptides is programmed in HF mice during fetal development.

We explored the critical role of AMPK by treating control NPCs with AMPK-specific modulators. In vitro pharmacologic inhibition of AMPK by Compound C mimicked the characteristics of HF NPC (i.e., decreased Mash1, Ngn3, POMC and increased AgRP). These findings are consistent with our hypothesis that reduced AMPK promotes AgRP versus POMC expression. The AMPK activator AICAR displayed results opposite to that of the Compound C. AICAR demonstrated increased Mash, Ngn3 and POMC with decreased AgRP expression. Together these findings confirm a critical role of AMPK on programmed hyperphagia. Future studies using genetically modified mice [59] would be essential to confirm the AMPK-mediated effects on NPCs. We speculate that in fetuses exposed to maternal obesity/high-fat diet and increased calorie levels, suppressed AMPK and neurogenic factors likely alters hypothalamic neurogenesis. The resulting increased appetite neurons contribute to offspring hyperphagia and obesity.

In HF offspring, the failure of leptin to suppress AMPK activity [60,61] suggests central leptin resistance. This is consistent with previous studies showing hyperleptinemia, central leptin resistance and upregulated AMPK activity in programmed obesity [34,62,63,64].

Among the various strains, C57BL/6J mice are the most widely used for high fat diet-induced obesity [65,66,67,68], as they exhibit abnormalities similar to human metabolic syndrome when fed a high-fat diet [69]. Given that C57BL/6 mice are genetically susceptible to diabetes and obesity, it is likely that this may influence the outcome [69]. However, few recent studies show similar weight gain in C57BL/6, A/J mice (resistant to obesity) and BALB/c mice on a high-fat diet [70,71]. Nonetheless, studies on strain-specific effects would provide a better understanding of the genetic influence on developmental programmed obesity.

In composite, these findings suggest that maternal obesity and high fat diet programs offspring obesity, in part via altered neurogenesis and programmed hyperphagia. The potential remains for additional mechanisms contributing to increased food intake. Epigenetic modifications have been implicated as a mechanism for changed expression of appetite/satiety neuropeptides. Studies show that neonatal overfeeding in mice caused hypermethylation of hypothalamic POMC promoter, which was consistent with its decreased expression [72]. Likewise, offspring exposed to maternal HF diet exhibited histone modification of POMC (decreased acetylation) and of NPY (increased acetylation). In humans, obese children had hypermethylated peripheral blood POMC gene as compared to normal weight children [73]. Notably, central reward pathway may also contribute to programmed hyperphagia as evident by increased preference for calorie-dense palatable foods in offspring exposed to maternal HF diet [74]. In addition, the development of neural connection within the central appetite pathway may be influenced by leptin, a hormone which reflects the level of adiposity.

## 5. Conclusions

In summary, consistent with animal studies and human epidemiologic data, these results demonstrate that maternal obesity and high fat-diet result in newborn macrosomia and offspring obesity. Enhanced food intake contributes significantly to accelerated growth and increased percent body fat. The increased appetite/satiety neuropeptide and ARC neuronal ratio suggests that an enhanced appetite leads to offspring hyperphagia. Our findings of altered AMPK and bHLH factors imply that an interactive pathway involving energy levels and neuroproliferation/neurodifferentiation is responsible for this programmed phenotype.

Although these results would suggest that hyperphagia and obesity are inevitable outcomes following pregnancy under these conditions, the potential exists for modification of upstream energy sensors (e.g., AMPK) via maternal diet or pharmacologic therapy, with a potential normalization of the fetal environment. In addition, the remarkable degree of postnatal ARC remodeling [75,76] offers the potential for similar newborn interventions to reverse an otherwise programmed effect. These findings provide a mechanistic basis for programmed hyperphagia and obesity and potential strategies that may target critical periods of pregnancy and/or newborn periods for the prevention of offspring obesity. Until additional data is available, the obese pregnant patient may be advised that pre-pregnancy weight loss, low energy diets, limited pregnancy weight gain, and exercise are likely to limit the programming of offspring obesity.

## Figures and Tables

**Table 1:** Maternal Body Weights.

	Control	HF
Body weight (g) at 3 weeks of age	10.2 ± 0.3	10.3 ± 0.2
Body weight (g) at 11 weeks	19.1 ± 1.1	25.0 ± 1.5 *
Body weight (g) at term	34.8 ± 0.5	36.8 ± 0.4 *
Body weight (g) at end of lactation	25.5 ± 0.3	27.9 ± 0.4 *
Litter Size	7.5 ± 0.5	6.9 ± 0.5

Body weights of female mice (*N* = 6 per group; mean ±SE) at ages 3 weeks (prior to high fat diet) and 11 weeks (at mating following control or high fat diet). Maternal body weights at term (e20) and at end of lactation (p21). Repeated measures of ANOVA; * *p* < 0.01 HF (high fat) vs Control.
